# Performance Analysis of CSMA/NP under Finite Population Environments

**DOI:** 10.3390/s24113290

**Published:** 2024-05-21

**Authors:** Ariadna I. Rodriguez-Gomez, Mario E. Rivero-Angeles, Izlian Y. Orea-Flores, Gina Gallegos-García, Juan Carlos Chimal-Eguia

**Affiliations:** Centro de Investigación en Computación, Instituto Politécnico Nacional, Mexico City 07738, Mexico; arodriguezg2020@cic.ipn.mx (A.I.R.-G.); erivero@cic.ipn.mx (M.E.R.-A.); ggallegos@cic.ipn.mx (G.G.-G.); chimal@cic.ipn.mx (J.C.C.-E.)

**Keywords:** access protocols, CSMA, finite population, throughput, WSNs

## Abstract

In this study, we analyze the CSMA Non-Persistent protocol with a finite number of nodes, providing more accurate results for applications like wireless sensor networks. The finite model addresses scenarios where the node count is moderate, capturing realistic system dynamics. Our analysis reveals a dependency on the node count, impacting system throughput. As the node count increases, throughput behavior aligns with Kleinrock’s infinite model. We derive a complex closed-form throughput expression for a finite quantity of nodes in the system, solved numerically, and offer an approximate expression for specific conditions. These insights advance understanding of low-contention network performance, especially in scenarios where the infinite model becomes inadequate.

## 1. Introduction

In modern communication systems, we encounter many possible scenarios where the number of nodes is relatively low compared, for example, to conventional cellular systems in a macro cell where the infinite population assumption is very accurate. For example, wireless sensor networks (WSNs) are systems composed of devices distributed within a defined area based on specific application requirements. In these systems, devices must support wireless communication, computation, and sensing capabilities [1,2,3]. There are many possible environments where WSNs are deployed, and, in some cases, the number of nodes contending to transmit in the shared channel may be relatively low. This occurs when nodes are sufficiently separated from each other (outdoor surveillance, for instance) or when a hierarchical system is used, for example, in clustered-based WSNs. In clustered-based WSNs, although hundreds of nodes might be present, the clustering scheme effectively reduces the number of nodes contending in each cluster, thereby simplifying the management of transmissions and potentially improving network efficiency. In these systems, nodes transmit data to a central base station using media access control protocols, among them, the Carrier Sense Multiple Access (CSMA) protocol [4].

The CSMA-Non-Persistent protocol (CSMA-NP) has been extensively studied in the literature [4,5,6,7,8], with a primary focus on scenarios involving an infinite population. However, there may be many circumstances where this is not the case. For instance, in the previously mentioned WSNs or even in 5G cellular systems, where carrier frequencies are in the region of gigahertz, producing very narrow areas of coverage. Additionally, this can occur in Internet of Things applications where only a couple of nodes are present. Therefore, there is a pressing need to analyze the CSMA-NP protocol within the context of a finite population assumption.

The analysis of a finite population within a communication network warrants careful consideration. Therefore, several authors, such as [4,5,6,7,8], have delved into this subject, exploring various scenarios that consider finite populations or retransmissions. The methodologies employed to assess network performance vary. These include the utilization of Markov chains, as seen in the work of [6]. Additionally, Monte Carlo simulations are employed, as demonstrated by [7]. Another approach is the application of the Exponential Backoff Scheduling algorithm, as demonstrated by [9]. The latter approach considers a system with multiple queues and a single server.

Despite the predominant focus on throughput when analyzing the performance of finite populations in the CSMA-NP protocol, much of the existing research has primarily focused on ad hoc networks. In contrast, the analysis presented in this paper considers a central base station. However, a significant gap remains in the current literature—much of the research on CSMA protocols, even when focused on finite node scenarios, is often based on protocol variants and not specifically on the CSMA-NP protocol itself. This oversight suggests a crucial need for dedicated studies that directly address the protocol in finite populations, offering insights that could be critical for the efficient design and management of modern network systems. Building on this, the main contributions of this work are as follows:

- We provide clear insights into the dynamics of the CSMA/NP random access protocol when the number of interfering nodes is low, which the infinite population model fails to consider. Specifically, in the vulnerability period, the number of interfering nodes reduces as the number of transmissions increases, whilst in the infinite population model, the packet transmission probability remains constant.
- We develop a closed-form expression to calculate the system throughput with N nodes. The vast majority of previous works consider an infinite population, which is accurate in WiFi, and cellular systems. However, in IoT applications, where only a few nodes interact directly (even if the network comprises hundreds of nodes, just the neighbor nodes interact due to the low transmission capabilities of the nodes in a WSN or due to the use of clustering schemes to provide energy savings, among others). We prove that our model is more accurate when the number of nodes is lower than 10, while the infinite population model provides adequate results for N > 10.
- We propose an approximated solution to simplify the throughput calculation. Indeed, we provide an exact expression for the throughput for the finite population scenario, but it has to be numerically solved. As such, we provide an approximation that does not require the numerical solution, but it provides a direct result that is accurate in certain regions. Hence, this approximation can be used instead of the numerical solution for the appropriate system variables’ range.

The remaining sections of this paper are structured as follows: In Section 2, we study the conventional CSMA-NP protocol under the infinite population model. Then, in Section 3, we introduce a novel approach to analyze the CSMA-NP protocol with a finite population assumption. In Section 4 some relevant results are presented, where we examine the system throughput using both a closed-form equation that we solve numerically and the simplified expression. We also analyze the implications of our findings when comparing them to the protocol with an infinite population. Finally, Section 5 concludes this paper and outlines potential avenues for future research.

## 2. CSMA-NP under the Infinite Population Assumption

The CSMA-NP protocol [10] has as its main objective to avoid collisions by rescheduling the packets when the sender node senses the channel is occupied. Conversely, if the channel is free, the node is cleared to send its packet.

This work develops an analysis that assumes an infinite population where the arrival process is considered to be a Poisson process with a packet arrival rate of *g* packets/s and each node sends only one packet at a time. Each of the packets is assumed to have a constant length of *T* seconds. When a collision occurs, the packets that collide turn into destructive interference and they cannot be decoded successfully. Also, a propagation delay of τ seconds is considered, which represents the time it takes a packet to be received by all contending nodes in the system. As such, after an idle period, when the transmitting node senses the channel, it will assume that it is free and start its transmission; if a second node attempts to transmit, it also senses the channel, but if this occurs before the first packet arrives, i.e., before τ seconds of the first transmission, this second node also senses the channel as idle and transmits its packet, generating a collision. If the second node senses the channel after τ seconds, then it senses the channel as occupied and alters its transmission, avoiding any collision. Hence, τ is usually referred to as the *vulnerability period*.

Thus, the throughput is the successful transmission rate $\bar{U}$ over the cycle of observation, which involves the expected duration of the channel in idle mode $\bar{I}$ and the expected duration of the channel in a busy period, whether a successful or packet collision occurs, $\bar{B}$ occurs. The analysis developed in [10] derives the following expression for the throughput (see (1) for further details):

(1) $$\begin{array}{cc} S & =\frac{\bar{U}}{\bar{I}+\bar{B}}=\frac{e^{-\tau g}}{\frac{1}{g}+T\left(1+\tau +\tau -\frac{\left(1-e^{-g\tau }\right)}{g}\right)}. \end{array}$$

However, this analysis assumes a constant arrival rate to the system (since an infinite population is considered), which is not accurate if the number of nodes is low. Indeed, as some nodes are allowed to transmit in the vulnerability period because they sensed the channel idle, the arrival rate drastically diminishes during the rest of that period, rendering a different system throughput. However, if the number of nodes is relatively high, the arrival rate remains almost constant irrespective of the number of previous transmissions. As such, the infinite population model renders accurate results for high values of *g*. Hence, we now study the system for a finite number of nodes in the following section.

## 3. CSMA-NP under the Finite Population Assumption

The finite population model (FP) assumes the same considerations as in the standardized protocol. Namely, that each node only transmits one package at a time and the packets take *T* seconds to be transmitted. However, the main difference falls in the arrival rate. As the number of nodes transmitting a packet increases, the remaining nodes that can transmit diminish, as opposed to the infinite population model where the arrival rate remains constant. To this end, consider that each node transmits a packet with rate α; then, the total arrival rate to the system is given by g=αN, where α represents the rate of arrival for each node, and *N* represents the nodes over the network.

**Figure 1:**
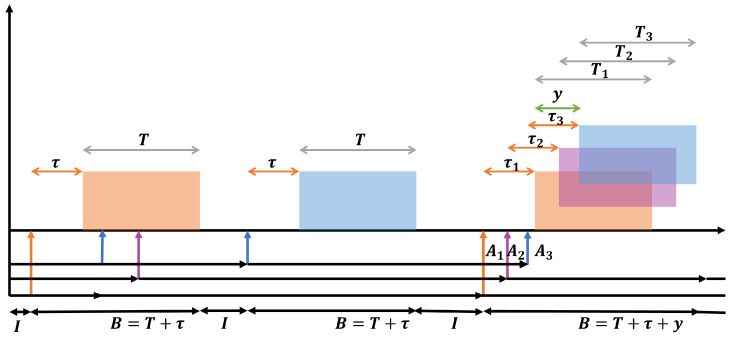
Non-persistent CSMA, Successful transmission and collision events.

In Figure 1, a network scenario with three nodes is depicted. From a top-down perspective, the third node attempts its initial packet transmission, highlighted in orange. Since the channel is unoccupied, the node begins to send the packet after a propagation delay of τ seconds, with the transmission taking *T* seconds. Meanwhile, the first and second nodes, upon sensing the channel, detect that it is occupied and consequently do not initiate transmission. Subsequently, after the third node has completed its transmission, the first node attempts to send a packet.

However, in a third scenario depicted, all three nodes attempt to send packets simultaneously. Each node, finding the channel idle after their respective vulnerability periods, initiates the transmission. These close transmissions lead to a collision. The duration of this collision spans from the first attempt to transmit to the point where the last node sends its packet. A transmission is deemed successful if it encompasses only the period from the intent to transmit to the conclusion of the transmission.

Building on this, the average time that the shared channel is useful can be obtained by observing that in case the packet is successful, it consumes *T* seconds of the channel, while it is 0 otherwise, i.e., no transmissions or packet collisions. Also, once a packet has been transmitted after an idle period, the probability that such a packet is successful, $P_{succ}$, is given by the probability that none of the remaining nodes, N−1, transmit in the vulnerability period, τ. Hence,

(2) $$\begin{array}{cc} P_{succ}=\; & Prob\{\mathrm{None}\;\mathrm{of}\;\mathrm{the}\;\mathrm{remaining}\;N\;-\;1\;\mathrm{nodes}\;\mathrm{transmit}\;\mathrm{in}\;\mathrm{the}\;\mathrm{vulnerability}\;\mathrm{period}\;\}\;\\ P_{succ}=\; & e^{-\alpha (N-1)\tau } \end{array}$$

From this, the average time that the system transmits useful information is given by

(3) $$\bar{U}=T*P_{succ}=T*e^{-\alpha (N-1)\tau }$$

To calculate the average time that the system is idle, we first calculate its cumulative distribution function (CDF), $F_{I}\left(t\right)=Prob\left(I\leq t\right)$.

(4) $$\begin{array}{cc} F_{I}\left(t\right)=\; & Prob(\mathrm{The}\mathrm{first}\mathrm{node}\mathrm{transmits}\mathrm{before}\tau )+\\ \; & Prob(\mathrm{The}\mathrm{second}\mathrm{node}\mathrm{transmits}\mathrm{before}\tau )+\\ \; & Prob(\mathrm{The}N^{\mathrm{th}}\mathrm{node}\mathrm{transmits}\mathrm{before}\tau )+\\ =\; & Prob(\mathrm{at}\mathrm{least}\mathrm{one}\mathrm{of}\mathrm{the}N\mathrm{nodes}\mathrm{transmit}\mathrm{before}\tau )\\ =\; & 1-\left[e^{-\alpha t}*e^{-\alpha t}*...*e^{-\alpha t}\right]=1-e^{-N\alpha t}, \end{array}$$

which is an exponential random variable with parameter Nα. Hence,

(5) $$\bar{I}=\frac{1}{N\alpha }$$

Finally, to calculate the average time that the system is busy, either by a successful packet transmission or packet collision, $\bar{B}$, can be calculated as follows: Whenever a packet transmission begins after an idle period, the system will remain busy for at least *T* seconds, which corresponds to the duration of the packet plus the propagation time, τ, plus the time after this first transmission and the last interfering packet that is transmitted before the vulnerability period, τ, ends. Let us call this time from the first transmission to the last interfering packet, *Y*. Then,

(6) $$\bar{B}=T+\tau +E\left[Y\right]$$

Since *T* and τ are constant values, we now concentrate on finding the mean value of *Y*. To this end, we first derive an expression for the CDF of the random variable *Y*, $F_{Y}\left(y\right)$. We can see that the probability that Y≤y is equivalent to the probability that there are no more arrivals in the vulnerability period after *y* seconds succeeding the first arrival, then $F_{Y}\left(y\right)=Prob\left(Y\leq y\right)=Prob\left(N\left(\tau -y\right)=0\right)$.

Where the function N(t) represents the number of arrivals in the period (0,t). Note that interfering nodes transmit before *y*; then, the non-interfering nodes have to transmit after τ, since they no longer collide with the rest of the packets. In this sense, this probability highly depends on the number of interfering nodes, $N_{I}$, because as the number of interfering nodes increases, the number of nodes that do not interfere decreases. This is the main issue in the finite population model that the finite population model cannot consider. As such, if there is only one interfering node, $N_{I}=1$, and considering that the first node that transmits is not an interfering node, then

(7) $$Prob\left(N\left(\tau -y\right)=0|n_{I}=1\right)=e^{-(N-2)\alpha (\tau -y)}$$

For the case of 2 interfering nodes:

(8) $$Prob\left(N\left(\tau -y\right)=0|n_{I}=2\right)=e^{-(N-3)\alpha (\tau -y)}$$

And in general, for *i* interfering nodes:

(9) $$Prob\left(N\left(\tau -y\right)=0|n_{I}=i\right)=e^{-(N-i-1)\alpha (\tau -y)}$$

As such, we find that:

(10) Prob(Y≤y)=∑i=1N−1N−1i1−e−αyie−N−1−iατ−y

From this, we find the expected value of $\hat{Y}$ as:

(11) Y^=∫0τ(1−FY(y))dy=∫0τ1−∑i=1N−1N−1i1−e−αyie−N−1−iατ−ydy=∫0τdy−∫0τ1−e−αy+e−ατ−yN−1dy−∫0τe−ατ−yN−1dy=τ−∫0τ1−e−αy+e−ατ−yN−1dy−e−α(N−1)τα(n−1).

Thus, by substituting (11) in the expression of $\bar{B}$, we get:

(12) $$\begin{array}{cc} \bar{B}= & T+2\tau -\int_{0}^{\tau }{\left(1-e^{-\alpha y}+e^{-\alpha \left(\tau -y\right)}\right)}^{N-1}dy-\frac{e^{-\alpha (N-1)\tau }}{\alpha (n-1)}. \end{array}$$

And finally, we derived the throughput equation presented in Equation (13). This equation is solved with a closed-form solution, which renders exact results but with high computational complexity that rapidly increases as the number of nodes in the system *N* increases. As such, we propose an approximated solution as we now describe.

(13) $$\begin{array}{c} \bar{S}\left(N\right)=\frac{Te^{-\alpha (N-1)\tau }}{\frac{1}{N\alpha }+T+2\tau -\int_{0}^{\tau }{\left(1-e^{-\alpha y}+e^{-\alpha \left(\tau -y\right)}\right)}^{N-1}dy-\frac{1}{\alpha (N-1)}e^{-\alpha (N-1)\tau }} \end{array}$$

### Approximated Solution

As we mentioned above, we obtained the system throughput for the FP model by a closed-form solution. However, for values of *N* higher than 10, it requires high computational resources to solve it. Hence, we derive an approximated method to solve this integral which results in a closed expression with very low complexity for any value of *N*, which allows us to easily evaluate the system throughput in any condition. To this end, the integral presented in (13) can be written as (14):

(14) $$\begin{array}{cc} \; & \int_{0}^{\tau }{\left(\left(1-e^{-\alpha y}\right)+\left(e^{-\alpha \left(\tau -y\right)}\right)\right)}^{N-1}dy=\int_{0}^{\tau }{\left(1-e^{-\alpha y}+e^{-\alpha \tau }e^{\alpha y}\right)}^{N-1}dy. \end{array}$$

Let x=αy and $a=e^{-\alpha \tau }$; then, $dx=\alpha dy\Rightarrow dy=\frac{1}{\alpha }dx$, substituting *x* and *a*, we get:

(15) $$\begin{array}{cc} \; & \int_{0}^{\alpha \tau }\;{\left(1\;-\;e^{-x}\;+\;ae^{x}\right)}^{\left(\;N\;-\;1\;\right)}\;\frac{1}{\alpha }\;dx\;=\;\frac{1}{\alpha }\int_{0}^{\alpha \tau }\;{\left(1\;-\;e^{-x}\;+\;ae^{x}\right)}^{N\;-\;1}dx. \end{array}$$

By considering $e^{x}=1+x+\frac{x^{2}}{2!}+\frac{x^{3}}{3!}+\frac{x^{4}}{4!}+...$, Equation (15) can be expressed as:

(16) $$\begin{array}{cc} \; & \frac{1}{\alpha }\;\int_{0}^{\alpha \tau }\;{\left(1\;-\;\left(\;1\;-\;x\;+\;\frac{x^{2}}{2!}\;-\;\frac{x^{3}}{3!}\;+\;\frac{x^{4}}{4!}\;-\;\theta \left(x^{5}\right)\right)\;+\;a\left(1\;+\;x\;+\;\frac{x^{2}}{2!}\;+\;\frac{x^{3}}{3!}\;+\;\frac{x^{4}}{4!}\;+\;\theta \left(x^{5}\right)\right)\;\right)}^{N-1}dx\\ \; & =\frac{1}{\alpha }\int_{0}^{\alpha \tau }{\left(x-\frac{x^{2}}{2!}+\frac{x^{3}}{3!}\;-\;\frac{x^{4}}{4!}\;+\;\theta \left(x^{5}\right)\;+\;a\;+\;ax\;+\;a\frac{x^{2}}{2!}\;+\;a\frac{x^{3}}{3!}+a\frac{x^{4}}{4!}\;+\;a\theta \left(x^{5}\right)\right)}^{N-1}dx. \end{array}$$

Reorganizing the terms, we obtain:

(17) $$\begin{array}{c} \frac{1}{\alpha }\;\int_{0}^{\alpha \tau }{\left(a\;+\;x\left(a\;+1\right)\;+\;\frac{x^{2}}{2}\left(a\;-\;1\right)\;+\;\frac{x^{3}}{3}\left(a\;+\;1\right)\;+\;\theta \left(X^{4}\right)\right)}^{N\;-\;1}\;dx. \end{array}$$

Let us consider a function such that:

(18) $$f\left(x\right)={\left(a+x\left(a+1\right)+\frac{x^{2}}{2}\left(a-1\right)+\frac{x^{3}}{3}\left(a+1\right)+\theta^{4}\right)}^{N-1}$$

And performing an expansion of Equation (18) around 0, we obtain:

(19) $$\begin{array}{cc} f(x)\;= & {\left(\;f\left(0\right)\;+\;f^{\prime }\left(0\right)\left(x-a\right)\;+\;\frac{f^{\prime \prime }\left(0\right){\left(x-a\right)}^{2}}{2}\;+\;\frac{f^{\prime \prime \prime }\left(0\right){\left(x-a\right)}^{3}}{3!}\;+\;...\right)}^{N-1} \end{array}$$

Note that:

(20) $$\begin{array}{cc} f(0) & =a^{N-1} \end{array}$$

(21) $$\begin{array}{cc} f^{\prime }\left(0\right) & =\left(N-1\right)a^{N-2}\left(a+1\right) \end{array}$$

(22) $$\begin{array}{cc} f^{\prime \prime }\left(0\right) & =a^{N-3}\left(N-1\right)\left(a^{2}\left(N-1\right)+a\left(2N-3\right)+\left(N-2\right)\right) \end{array}$$

And by considering Equation (19), a Taylor series can be constructed by substituting with (20), (21) and (22):

(23) $$\begin{array}{cc} f(x) & =a^{N\;-\;1}\;+\;\left(N\;-\;1\right)a^{N\;-\;2}\left(a\;+\;1\right)x\;+\;\\ \; & a^{N\;-\;3}\left(N\;-\;1\right)\left(a^{2}\;\left(N\;-\;1\right)\;+\;a\;\left(2N\;-\;3\right)\;+\;\left(N\;-\;2\right)\right)\;\frac{x^{2}}{2}\;+\;... \end{array}$$

And by substituting in (17) and organizing the terms, we obtain:

(24) $$\begin{array}{c} \begin{array}{cc} \; & \frac{1}{\alpha \tau }\int_{0}^{\tau }(a^{N-1}+\left(N-1\right)a^{N-2}\left(a+1\right)x+\\ \; & a^{N-3}(\left(N-1\right)\left(a^{2}\left(N-1\right)+a\left(2N-3\right)+\left(N-2\right)\right)\frac{x^{2}}{2}+\theta \left(x^{3}\right)\frac{x^{3}}{3}...)dx\\ =\; & \eta \end{array} \end{array}$$

(25) $$\begin{array}{c} \begin{array}{cc} \eta = & \frac{1}{\alpha }[a^{N-1}x+\left(N-1\right)a^{N-2}\left(a+1\right)\frac{x^{2}}{2}+\\ \; & \frac{1}{2}a^{N-3}\left(N-1\right)\left(a^{2}\left(N-1\right)+a\left(2N-3\right)+\left(N-2\right)\right)\frac{x^{3}}{3}+\theta \left(x^{4}\right)]\;\;{|}_{0}^{\alpha \tau } \end{array} \end{array}$$

Then, we consider the original values of x=αy and $a=e^{-\alpha \tau }$, and after some algebraic manipulation, we find:

(26) $$\begin{array}{c} \begin{array}{cc} \eta =\; & e^{-\alpha \tau (N-1)}\alpha \tau +\left(N-1\right)e^{-\alpha \tau (N-2)}\left(e^{-\alpha \tau }+1\right)\frac{{\left(\alpha \tau \right)}^{2}}{2}+\\ \; & \frac{1}{6}e^{-\alpha \tau (N-3)}\left(N-1\right)(e^{-{\left(\alpha \tau \right)}^{2}}\left(N-1\right)+e^{-\alpha \tau }\left(2N-3\right)+\\ \; & {\left(N-2\right))\left(\alpha \tau \right)}^{3}+\theta \left(\tau^{4}\right) \end{array} \end{array}$$

(27) $$\begin{array}{c} \begin{array}{cc} \;\int_{0}^{\alpha \tau }{\left(1-e^{-\alpha y}+e^{-\alpha (\tau -y)}\right)}^{N-1}dy= & e^{-\alpha \tau (N\;-\;1)}\alpha \tau \;+\left(N\;-\;1\right)e^{-\alpha \tau (\;N\;-\;2\;)}\left(e^{-\alpha \tau }\;+\;1\right)\frac{{\left(\alpha \tau \right)}^{2}}{2}+\\ \; & \frac{1}{6}e^{-\alpha \tau (N-3)}\left(\;N\;-\;1\;\right)(e^{-2\alpha \tau }\left(\;N\;-\;1\;\right)+\\ \; & e^{-\alpha \tau }{\left(2N-3\right)+\left(N-2\right))\left(\alpha \tau \right)}^{3}+\theta \left(\tau^{4}\right) \end{array} \end{array}$$

Finally, let $e^{-\alpha \tau }$ be *a*, and by substituting (27) in (13), we obtain (28).

(28) $$\begin{array}{cc} \; & \;\bar{S}\left(N\right)\;=\;\frac{\bar{U}\left(N\right)}{\bar{I}\left(N\right)+\bar{B}\left(N\right)}\\ \; & \;=\;\frac{Ta^{\left(N\;-\;1\right)}}{\frac{1}{N\alpha }+T\;+\;2\tau \;-\;\alpha \tau \;(\;a^{\left(N\;-\;1\right)}\;+\;\left(N\;-\;1\right)a^{\left(N\;-\;2\right)}\left(a\;+\;1\right)\frac{\alpha \tau }{2}\;+\;\frac{1}{6}a^{\left(N\;-\;3\right)}\left(N\;-\;1\right)\left(a^{2}\left(N\;-\;1\right)\;+\;a\left(2N\;-\;3\right)\;+\;\left(N\;-\;2\right)\right){\left(\alpha \tau \right)}^{3}\;-\;\frac{a^{\left(N\;-\;1\right)}}{\alpha (N\;-\;1)}} \end{array}$$

To evaluate the performance of the proposed closed-form and the approximated solution, we develop a system simulation presented in the pseudocode Algorithm 1:
**Algorithm 1:** Simulation System
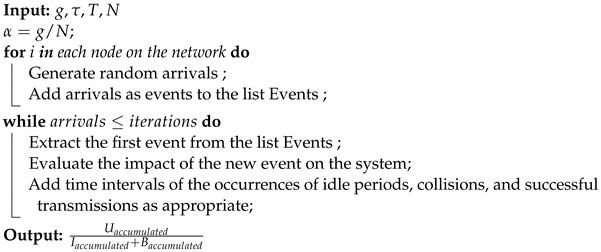


The system simulation is summarized in Algorithm 1, which operates based on the discrete event paradigm. Given the input variables g,τ,T,N, and assuming that α=g/N, the algorithm initiates by generating a first attempt to send a packet for each network node. Subsequently, these packet arrival events are sorted in chronological order. The simulation then proceeds iteratively; the first element of the sorted list is processed based on the system’s current state.

The algorithm evaluates the impact of each new event on the current state of the system. Possible events include arrivals, end of transmission, and end of the vulnerability period. The variables that accumulate the time intervals for different system modes are:

- $U_{accumulated}$: The increments are T+τ for each successful transmission.
- $I_{accumulated}$: Records the time intervals from the last end of transmission until a new arrival; the increments are not standardized.
- $B_{accumulated}$: Updates with the time interval from the first arrival until the last arrival has completed its packet transmission. Note that the last arrival to send a packet occurs between the first arrival and the end of the vulnerability period of the first arrival, so the increment should be within T+τ and T+2τ.

For example, if the channel is occupied with a single transmission and a new event is an arrival, it will not be taken into account. The cycle then continues to the next event on the list without making any changes. At the end of the current transmission, the variables $U_{accumulated}$ and $B_{accumulated}$ are increased by T+τ, as the channel has been busy and a successful transmission occurred.

Conversely, if an attempt to transmit information occurs before the vulnerability period has finished and the new event is an arrival, the new arrival causes a collision. In this case, $B_{accumulated}$ is updated with the time interval from the first arrival until the last arrival has sent its complete packet, representing the time during which the channel has been busy. The $I_{accumulated}$ variable records the time lapses from the last end of transmission until the first arrival, indicating that the channel has been in idle mode.

Finally, the output should be the result of $\frac{U_{accumulated}}{I_{accumulated}+B_{accumulated}}$, which corresponds to the throughput equation in Equation (1). The algorithm should ensure that the iterative process is repeated sufficiently to achieve steady-state results. In this particular case, we performed 100,000 iterations, which was a value founded based on empirical observations and testing by running the algorithm with the same variables several times.

We developed our simulator in Python due to its versatility and extensive library support. Our simulator is based on the methods mentioned above and was implemented without the use of additional simulation packages. Python’s robust libraries and tools enabled us to efficiently manage the complex calculations and event-driven processes required for our simulation.

## 4. Numerical Results

**Figure 2:**
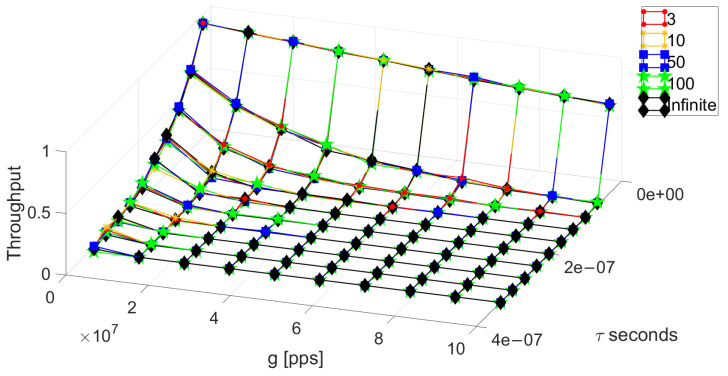
Throughput simulation of the system with 3, 10, 5, 100 and an infinite number of nodes.

In this section, we present the most relevant results derived from this work. In addition to the analysis developed above, we performed multiple system simulations to validate the analytical results, for both the exact and approximated throughput expressions. We first obtain the system throughput using Equation (13) and compare it to the results under the infinite population model; this infinite model considers that the packet arrival rate remains constant. Conversely, in our proposed model, the rate of packet transmissions decreases according to the number of packets previously transmitted, which renders different, more accurate results for low values of N. In Figure 2, we show the throughput for different values of *N* for the finite population model, varying the arrival rate (*g*) from $1\times 10^{-7}$ to $1\times 10^{-6}$ packets per second (pps) and the vulnerability time (τ) from $3.3\times 10^{-8}$, taking the speed of light in a vacuum and considering a distance between the sender and receiver of 10 m, to $3.3\times 10^{-7}$ [seconds] (100 m), the *T* value is fixed to 0.001. We can see that the FP model differs greatly from the infinite population model when *N* is low and when the packet arrival rate is low, i.e., for low values of *g* and τ. Note that the infinite population model provides a very good approximation to the system dynamics when the traffic load remains relatively constant during the vulnerability period. This occurs for high values of *g* and τ, but for many IoT applications where packet transmissions are scarce, our proposed model better captures the behavior of the system.

**Figure 3:**
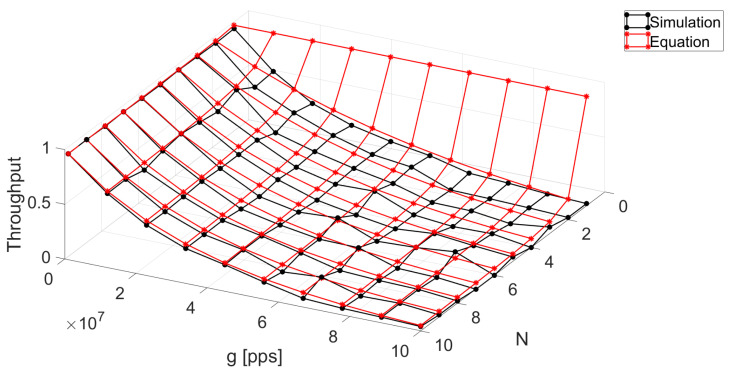
Comparison between the simulation of the system, and the closed-form solution for an FP with $\tau =\left(3.33\right)\times 10^{-8}$ and T=0.001.

Then, we validate our analysis by comparing the results of the closed-form (red surface) of the throughput under the finite population model to the system simulations (black surface), as depicted in Figure 3. We can see a very good match between these results and a very slight difference when a single node is in the system producing almost no packet transmissions.

**Figure 4:**
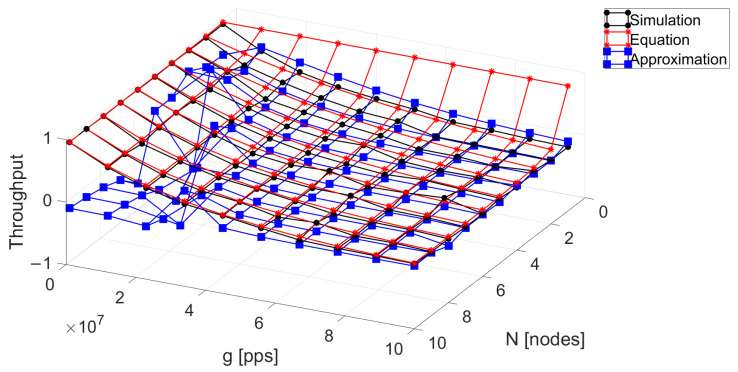
Comparison between the simulation of the system, the closed-form solution, and the numerical method for an FP with τ=0.1 and T=1.

We now evaluate the accuracy of the proposed approximation (blue surface) to the closed-form expression of the system throughput under the finite population model. We can see in Figure 4 that, in general, the proposed approximation presents a very good match compared to the closed expression of the throughput (red surface) and the system simulation (black surface). Although some points present a higher discrepancy, we believe that it still accurately captures the system behavior with the great advantage that it provides results for any value of *N* with low computational complexity, while the closed exact expression requires numerically solving the integral, adding some inconvenient complexity. To assess the precision of the approximation, we calculate the relative error, $RE=\sum_{\mathrm{For}\;\mathrm{all}\;i}\frac{\left(x_{i}-{\hat{x}}_{i}\right)}{x_{i}}$, where $x_{i}$ represents the values obtained through the simulation and it acts as the reference and ${\hat{x}}_{i}$ are the values obtained through the approximation solution. It is crucial to note that the optimal application scenario for this approximation occurs when *g*, defined as αN, exceeds three times 1/τ.

The relative error between the closed expression and the proposed approximation is 0.4874. It can be observed that the regions where there are the most discrepancies are when the traffic offered is below fifty percent. The rationale behind this is that the terms of the Taylor series that are ignored have an important impact on the calculation of the arrival of the interfering packets *Y*. We believe that including additional terms to this approximation may result in a better match but with an increased complexity. For practical effects, the proposed approximation renders a good match in most of the system parameters and we leave the study of a better approximation to future work since we believe that it lies outside the scope of this paper.

The main advantage of the proposed finite population model is that it provides more accurate results when the number of interfering nodes is reduced. Specifically, when the nodes in the transmission range are low, when some nodes have previously transmitted their packet, the remaining nodes that can transmit are reduced accordingly, which is not considered in an infinite population model. This model would render more accurate results in the context of WSN and IoT applications, where, even if the number of nodes is high, due to the low transmission capabilities of the nodes (usually 10 to 50 m compared to hundreds of meters in a cellular system), only a handful of them can interfere, or even in cases where clustering and ON/OFF schemes are used, drastically reducing the number of interfering nodes in the surroundings. We believe that our model can be used in energy consumption calculation or cyber-attack detection using more accurate results than the infinite population model. However, this analysis renders a complex equation with an integral that can only be numerically solved. As such, we provide an approximation that does not require the numerical solution of the integral, but this method is only adequate when it meets the previously mentioned relationship g>3/τ.

## 5. Conclusions

In this work, we studied, analyzed, and evaluated the CSMA-NP protocol for environments where the number of nodes is low or the packet arrival rate depends on the number of active transmissions. Such environments include many modern communication systems that the infinite population model cannot capture accurately. We derive a closed expression of the system throughput but it includes an integral that has to be numerically solved. Hence, we also propose an approximation that presents a good fit in most cases and with low computational complexity. We validated our results with system simulations.

As expected, the infinite population model renders very different throughput results when there are a few nodes in the network and the packet arrival rate is low. This would generate important discrepancies in practical environments in terms of packet delay and energy consumption if the infinite population model is used in certain system conditions. Conversely, when the number of nodes is high, greater than N=10, the infinite population model accurately captures the system performance since the packet arrival rate in the vulnerability period does not greatly vary anymore.

Overall, this study highlights the importance of considering finite populations in the analysis of the CSMA-NP protocol and provides valuable insights into how the system behaves as the number of nodes varies and offers a proposed equation that, while generally accurate, may require adjustments for specific scenarios. These findings contribute to a deeper understanding of network performance and open the door to future research directions for refining the proposed equation and extending the analysis to different network configurations and scenarios.

## Data Availability

Data are contained within the article.
